# Pleural solitary fibrous tumour with brain metastasis: an aggressive tumour and pathologic conundrum

**DOI:** 10.1093/jscr/rjac344

**Published:** 2022-08-04

**Authors:** Catherine Williams, Daniel French, Sean Christie, Mathieu Castonguay, Alison Wallace

**Affiliations:** Department of Surgery, Division of Thoracic Surgery, Queen Elizabeth II Health Sciences Centre, Dalhousie University, Halifax, Nova Scotia, Canada; Department of Surgery, Division of Thoracic Surgery, Queen Elizabeth II Health Sciences Centre, Dalhousie University, Halifax, Nova Scotia, Canada; Department of Surgery, Division of Neurosurgery, Queen Elizabeth II Health Sciences Centre, Dalhousie University, Halifax, Nova Scotia, Canada; Department of Pathology, Queen Elizabeth II Health Sciences Centre, Dalhousie University, Halifax, Nova Scotia, Canada; Department of Surgery, Division of Thoracic Surgery, Queen Elizabeth II Health Sciences Centre, Dalhousie University, Halifax, Nova Scotia, Canada; Department of Pathology, Queen Elizabeth II Health Sciences Centre, Dalhousie University, Halifax, Nova Scotia, Canada

## Abstract

Solitary fibrous tumours of the pleura are a rare finding; those with brain metastases are rarer still. Here, we present the evolution of a pleural solitary fibrous tumour in a 70-year-old male treated surgically, and subsequent brain metastasis requiring emergent craniotomy and excision. The patient received adjuvant radiotherapy to the brain and had no recurrence of brain metastases; however, 1 year surveillance imaging demonstrated metastases to the lungs, liver and spleen for which he received chemotherapy but eventually succumbed to the disease process. Solitary fibrous tumours are most often slow-growing, relatively benign neoplasms. However, up to 10% are malignant. This case highlights the importance of surgical resection of these benign tumours with malignant potential.

## INTRODUCTION

Solitary fibrous tumours (SFTs) are rare mesenchymal neoplasms that can occur anywhere in the body but are most often found in the pleura or lungs [1]. Pleural SFTs have a low incidence of metastasis to the brain [2], with few cases reported in the literature [3]. This is a case report of a 70-year-old man with a left-sided pleural SFT who underwent surgical resection of both the pleural mass and of a solitary brain metastasis followed by adjuvant treatment.

## CASE REPORT

**Figure 1:**
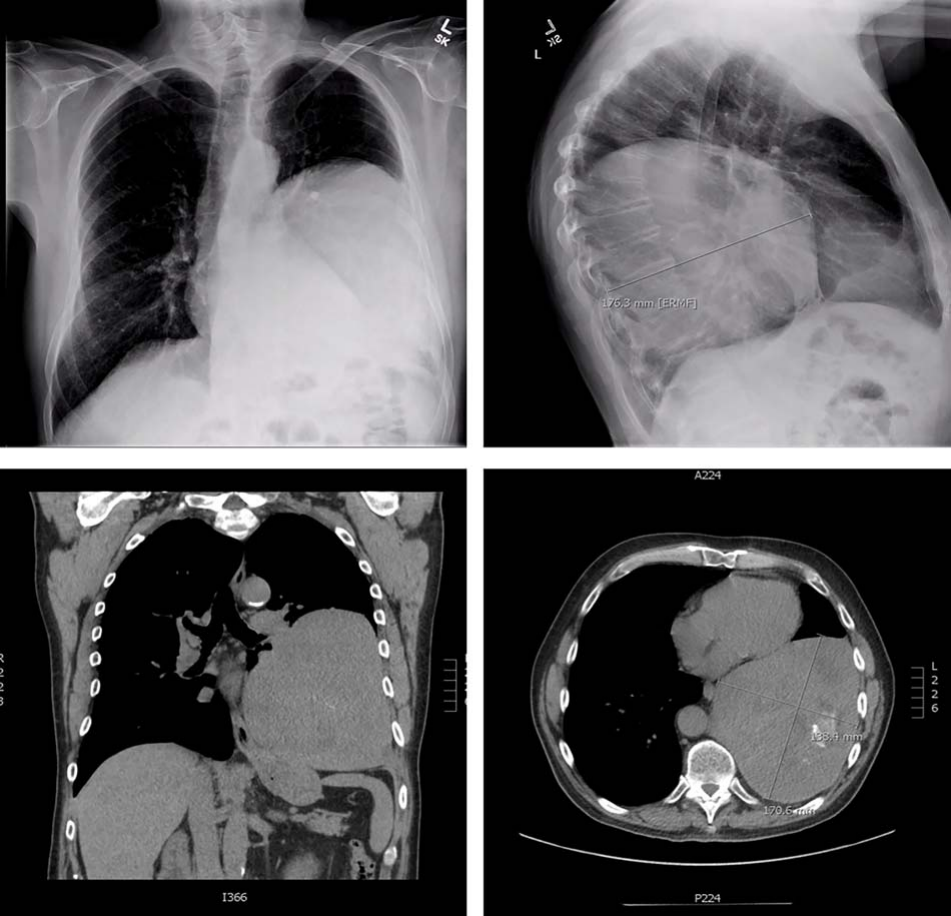
Representation of solitary fibrous tumour in 2019 showing 18.3 × 17.0 × 13.8 cm left-sided pleural-based tumour.

**Figure 2:**
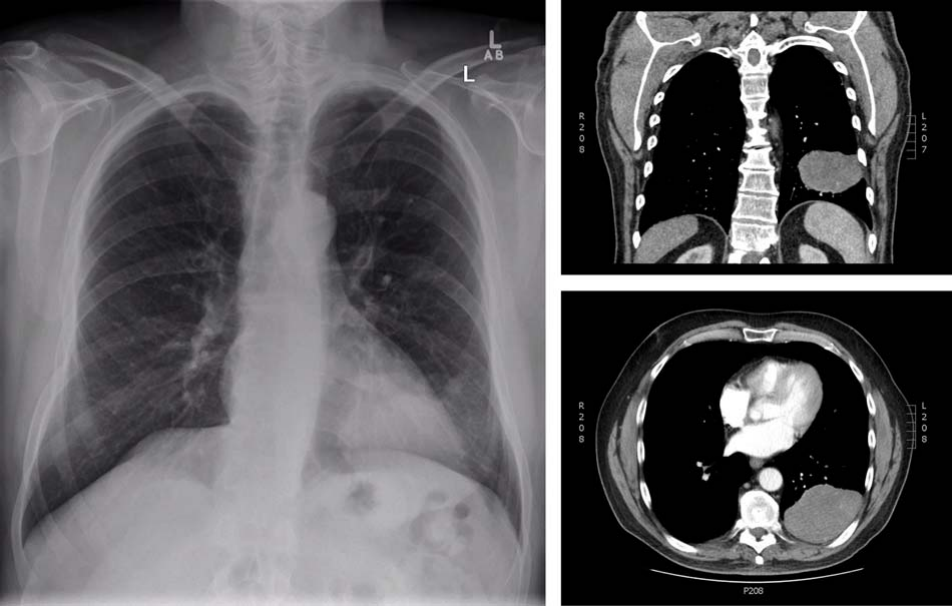
Initial presentation of solitary fibrous tumour in 2013 showing 9.3 × 6.1 cm left-sided pleural-based tumour.

The patient is a 70-year-old man with a 75-pack-year smoking history and occupational asbestos exposure, who presented with back pain and constitutional symptoms in 2019. Physical exam was unremarkable except for hypertrophic pulmonary osteoarthropathy with digital clubbing. Computed tomography chest demonstrated a 18.3 × 17.0 × 13.8 cm left pleural-based tumour, with heterogeneous density and internal calcifications, resulting in compression of the left lower bronchi (Fig. 1). The patient’s medical records revealed that he was originally diagnosed with a left-side pleural SFT in 2013 (Fig. 2); imaging and biopsy at that time revealed a 9.3 cm spindle cell tumour consistent with SFT. The patient was lost to follow-up.

**Figure 3:**
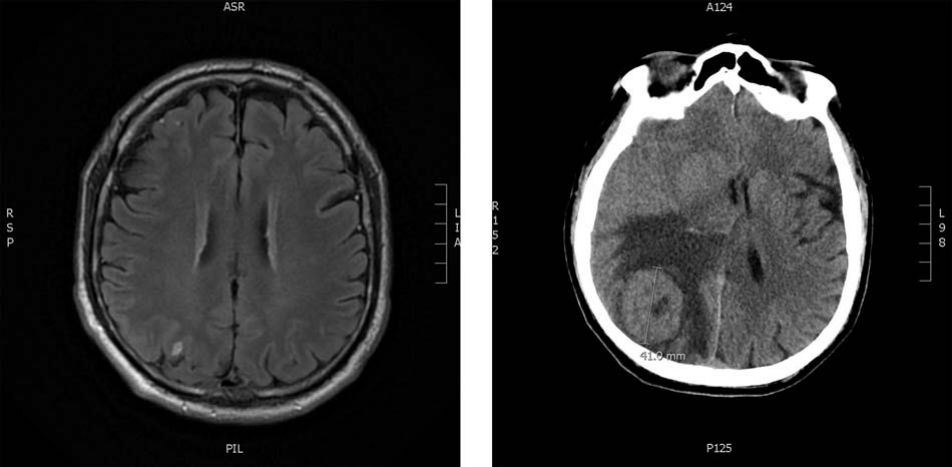
Brain imaging; solitary right parietal brain metastasis measuring 6 mm August 2019 (left) and 4.5 months later measuring 4.1 cm (right).

**Figure 4:**
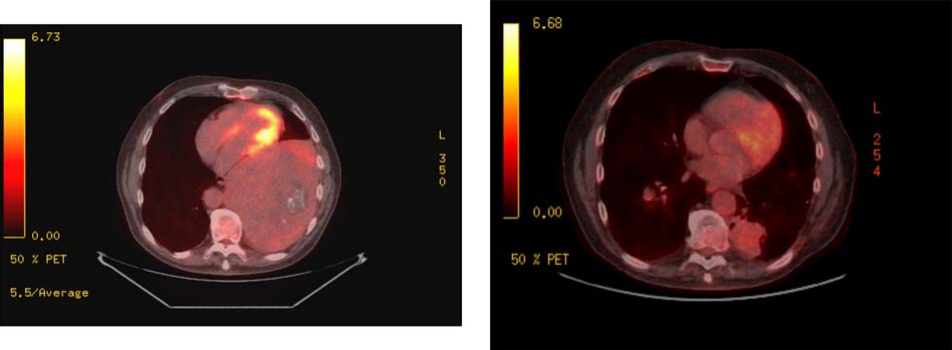
PET scans; large left-sided pleural-based tumour exhibits mild FDG uptake, with the SUV max of 2.9 (left); recurrent disease at the pulmonary resection margin along with contralateral pulmonary metastases demonstrated (right).

**Figure 5:**
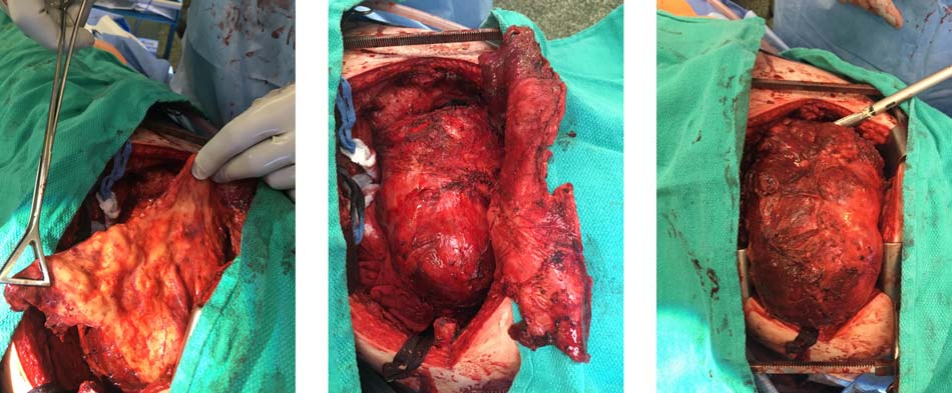
Gross appearance of solitary fibrous tumour during *en bloc* resection via left thoracotomy showing thickened pleural and tumour *in situ*.

Upon re-presentation in 2019, workup included brain magnetic resonance imaging (MRI), positron emission tomography (PET) scan and re-biopsy. Brain imaging revealed a 6 mm solitary right parietal brain metastasis (Fig. 3). On PET scan, the tumour exhibited mild FDG uptake, with the SUV max of 2.9 (Fig. 4). There was no evidence of nodal or distant metastatic disease. Biopsy again was consistent with SFT. The patient was reviewed at Multidisciplinary Thoracic Tumour Board Rounds. Considering the large size and compressive effects of the pleural-based tumour, combined with the small size and asymptomatic nature of the brain lesion, it was determined that the optimal approach would be surgical resection of the left-sided pleural SFT and active surveillance of the brain metastasis. The patient underwent complete *en bloc* resection of the tumour via left thoracotomy including the eighth rib, partial pleurectomy and a left lower lobe wedge resection to achieve an R0 resection (Fig. 5).

**Figure 6:**
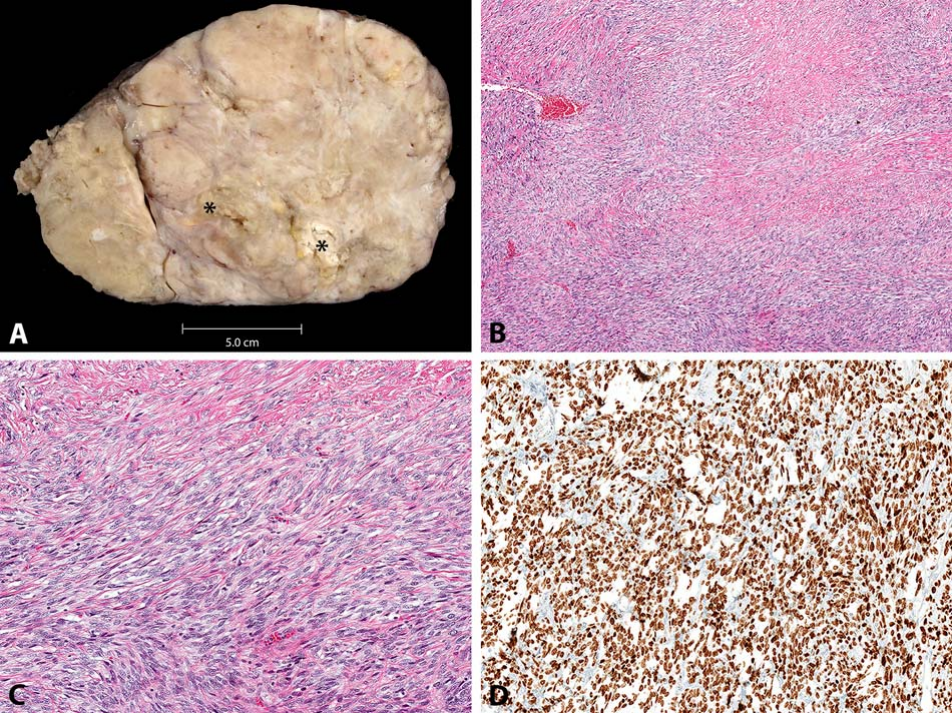
**A**, macroscopic appearance of solitary fibrous tumour, with foci of necrosis (*). **B**–**D**, histologic appearance of solitary fibrous tumour, with variably cellular bland spindled cells in vague fascicles and with diffuse STAT6 immunoreactivity (B, H&E stain, original magnification x40; C, H&E stain, original magnification x100; D, STAT6 immunohistochemical study, original magnification x100).

**Figure 7:**
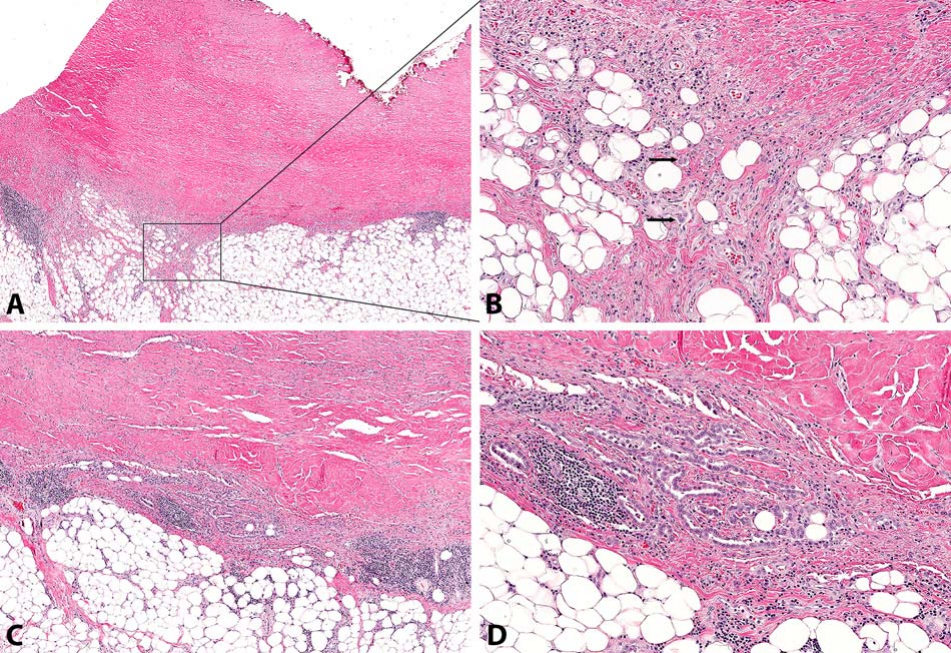
Atypical mesothelial proliferation composed of single cells and small cell clusters (**A**&**B**) and irregular tubules (**C**&**D**), with invasion of subpleural adipose tissue (arrows, B) (A, H&E stain, original magnification x20; B, H&E stain, original magnification x100; C, H&E stain, original magnification x40; D, H&E stain, original magnification x100).

Pathology from the case demonstrated two significant findings. The left pleural-based tumour demonstrated uniform spindle cells growing in haphazard, fascicular and storiform patterns, with up to four mitotic figures per 10 high-power fields and ~5% tumour necrosis; there was focal invasion of the left lung, and immunohistochemical reactivity to STAT6, CD34 and BCL-2 antibodies (Figs 6 and 7). The overall features were those of an SFT with a high metastatic potential, according to the risk stratification model proposed by Demicco *et al.* [4]. In addition, the left parietal pleura overlying the SFT contained an atypical mesothelial proliferation suspicious for diffuse epithelioid mesothelioma; however, these mesothelial cells retained immunohistochemical expression of BAP1 and MTAP, and the possibility of an unusual reactive mesothelial proliferation induced by the SFT could not be excluded. The consensus at Thoracic Multidisciplinary Tumour Board Rounds was reactive mesothelial cells as opposed to mesothelioma and adjuvant chemotherapy was not recommended.

Approximately 1 month after discharge from the initial procedure, the patient presented acutely with left hemiplegia. MRI of the head demonstrated significant growth of the right parietal lesion, now 4.1 × 4 × 3.5 cm, with new surrounding edema (Fig. 3). The patient underwent a right parietal craniotomy with gross total resection of the lesion and adjuvant radiotherapy to the brain (60 Gy in 30 fractions). Pathology confirmed the diagnosis of metastatic SFT.

The patient was followed closely with surveillance scans every 3 months. One year later, the patient regained 95% of his neurologic function and there was no evidence of further brain lesions; however, at that time, recurrent disease was detected at the pulmonary resection margin along with bilateral pulmonary nodules, liver and splenic metastases. The patient was treated with radiation to the recurrence at the pulmonary resection margin and initially palliative chemotherapy with Doxorubicin and Dacarbazine and was later started on Sunitinib.

Six months later, the tumour further metastasized to the right gluteus muscle and right rectus femoris muscle. Tumour thrombosis developed within the left renal vein and extended into the hepatic and infrahepatic part of the IVC. The patient died due to complications related to the disease shortly after in 2021.

## DISCUSSION

This case highlights that although the majority of SFTs behave in an indolent fashion, some tumours exhibit more aggressive behaviour and complete *en bloc* surgical resection remains the mainstay of treatment. In a public health care system, this case provides a unique look into the natural history of pleural-based SFTs and provides insight into the metastatic potential of these tumours.

## CONFLICT OF INTEREST STATEMENT

None declared.
